# Introduction of weekly multidisciplinary teammeetings in a tertiary cardiothoracic critical care unit: evaluation of staff satisfaction and impact on clinical outcomes

**DOI:** 10.1186/2197-425X-3-S1-A862

**Published:** 2015-10-01

**Authors:** S Gelvez-Zapata, R D'Oliveiro, K Eriksson, J Bracken, M Pontin, N Jones

**Affiliations:** Papworth Hospital, Intensive Care, Cambridge, United Kingdom; Papworth Hospital, Intensive Care, Cambridge, United Kingdom

## Introduction

The need for improved the quality of patient care in the intensive care unit (ICU) make it imperative to analyse the effects of the introduction of a multi-disciplinary team meetings (MDT) and its benefits in other areas.

## Objectives

To evaluate the impact of a weekly MDT in a specialist cardiothoracic ICU on selected indicators of quality of care and assess staff perception since the MDT implementation.

## Methods

A survey was designed and distributed among the ICU professionals involved in patient care at Papworth Hospital. The surveys were distributed on paper or electronically and responses collated between September 29^th^ and October 27^th^ 2014. In total, 11 multiple-choice and open questions were used in the survey. We compared the rates of four indicators of quality of care, out of hours discharges, repatriations, unplanned extubations and readmissions, for 18 months before and 5 months after the survey. The results were presented to the Intensive Care Department as Phase I of the study on 25^th^ February 2015, and conclusions were used to implement Phase II, which is currently in process.

## Results

87 people participated in the survey, of whom 58% were nurses and 12% doctors. 81% of the professionals were aware of the weekly MDT meeting but just 29% were able to attend at least once a month. More than 50% of the professionals felt that they could contribute to the MDT and 75% thought that MDTs had a positive impact on patient care, but there were a number of aspects that needed to improve to maximise this such as encourage staff to attend MDT meetings, immediate communication and implementation of decisions taken during the meeting and discussed on daily ward rounds and during bedside handovers. The survey revealed that the patients and their relatives did not have a voice in the MDT. Following the survey we found that rates of out of hours discharges decreased by 50% (p < 0.06; Poisson test), readmissions reduced by 35% (p < 0.07; Poisson test), repatriations increased by 27% (p < 0.1; Poisson test) and unplanned extubations reduced by 68% (ns).

## Conclusions

The weekly ICU MDT appears to have had a positive impact on patient care. Staff felt their input was valued. However the survey highlighted areas for improvement, in particular ensuring greater involvement of staff, patients and relatives. We observed some improvements in out of hours discharges, readmissions and repatriations following the survey. The results of this study will be applied in Phase II and it will be reaudit following implementing changes.

## References

[1] Sandham D (1988). The value of a multi-disciplinary team in critical care. Can Fam Physician.

[2] Cheung W, Millis D, Thhanakrishnan G, Anderson R, Tan JT (2009). Effect of implementation of a weekly multidisciplinary team meeting in a general intensive care unit. Crit Care Resusc.

